# MARCH3 negatively regulates IL-3-triggered inflammatory response by mediating K48-linked polyubiquitination and degradation of IL-3Rα

**DOI:** 10.1038/s41392-021-00834-7

**Published:** 2022-01-24

**Authors:** Lu Feng, Chen Li, Lin-Wen Zeng, Deng Gao, Yu-Hao Sun, Li Zhong, Heng Lin, Hong-Bing Shu, Shu Li

**Affiliations:** Department of Infectious Diseases, Zhongnan Hospital of Wuhan University, Medical Research Institute, Frontier Science Center for Immunology and Metabolism, Research Unit of Innate Immune and Inflammatory Diseases, Chinese Academy of Medical Sciences, Wuhan University, 430071 Wuhan, China

**Keywords:** Immunological disorders, Inflammation

## Abstract

Interleukin-3 (IL-3) is a hematopoietic growth factor and critical regulator of inflammatory response such as sepsis. IL-3 binds to IL-3 receptor α (IL-3Rα), which is then associated with IL-3Rβ to initiate signaling. How IL-3-triggered physiological and pathological effects are regulated at the receptor level is unclear. Here, we show that the plasma membrane-associated E3 ubiquitin ligase MARCH3 negatively regulates IL-3-triggered signaling. MARCH3 is associated with IL-3Rα, mediates its K48-linked polyubiquitination at K377 and promotes its proteasomal degradation. MARCH3-deficiency promotes IL-3-triggered transcription of downstream effector genes and IL-3-induced expansion of myeloid cells. In the cecal ligation and puncture (CLP) model of sepsis, MARCH3-deficiency aggravates IL-3-ampified expression of inflammatory cytokines, organ damage and inflammatory death. Our findings suggest that regulation of IL-3Rα by MARCH3 plays an important role in IL-3-triggered physiological functions and inflammatory diseases.

## Introduction

Interleukin-3 (IL-3) is a hematopoietic growth factor that is mainly secreted by activated T-cells. IL-3 plays an important role in the development of multi-lineage cell types including granulocytes, monocytes, macrophages, eosinophils, basophils, and mast cells.^1^ IL-3 also serves as a growth factor for leukemic colonies with an elevated IL-3 receptor α-chain (IL-3Rα, CD123) such as acute myeloid leukemia, chronic myelogenous leukemia or plasmacytoid dendritic cell neoplasm.^2,3^ It has been demonstrated that IL-3 amplifies acute inflammation in murine sepsis, and elevated plasma levels of IL-3 are associated with high mortality in human sepsis.^4^ Recently, it has been shown that the astrocyte-sourced IL-3 programs microglia to ameliorate the pathology of Alzheimer’s disease in human and mice.^5^

Signaling of IL-3 is initiated by its binding to IL-3Rα. This triggers association of IL-3Rα with IL-3 receptor β-chain (IL-3Rβ, CD131), a common receptor subunit also for granulocyte–macrophage colony-stimulating factor (GM-CSF) and IL-5.^6,7^ Binding of IL-3 to its receptor complex recruits and activates receptor-associated Janus kinases (JAKs), predominantly JAK2.^8^ The activated JAK2 then recruits the transcription factor signal transducer and activator of transcription 5 (STAT5) and mediates its phosphorylation at Y694/699.^9,10^ Phosphorylated STAT5 translocates into the nucleus to induce transcription of a set of downstream effector genes. In addition to the JAK/STAT pathway, IL-3 also activates Ras-MAPK and phosphoinositol 3-kinase (PI3K) pathways. These pathways collectively lead to diverse biological and pathological responses.^6,11–13^ Studies have shown that IL-3-triggered signaling is regulated by various mechanisms. The protein-tyrosine phosphatase SHP2 negatively regulates IL-3-driven cell survival and proliferation via regulation of tyrosine phosphorylation of STAT5.^14^ The hematopoietic GTPase RhoH negatively modulates IL-3 signaling through regulation of STAT activity and IL-3Rα expression.^15^ These studies emphasize the need for tight control of IL-3/IL-3Rα axis in various physiological and pathological processes.

The membrane-associated RING-CH-type finger (MARCH) proteins are E3 ubiquitin ligases that have emerged as critical regulators of immune receptors, viral proteins, and membrane-associated components.^16,17^ Except for MARCH7 and MARCH10, the majority of 11 mammalian MARCH proteins share a similar structure, including an N-terminal RING-CH finger and two or more TM domains, which distinguish them as membrane-associated E3 ubiquitin ligases. The MARCH family contains four well-defined subgroups including MARCH1 and 8, MARCH2 and 3, MARCH7 and 10, and MARCH4, 9 and 11.^16,17^ Recently, certain MARCH proteins have been identified as important regulators of immune receptors. For example, it has been shown that MARCH3 attenuates IL-1β-triggered inflammation by mediating K48-linked polyubiquitination and degradation of IL-1 receptor I (IL-1RI),^18^ whereas MARCH8 targets the IL-1 receptor accessary protein (IL-1RAcP) for K48-linked polyubiquitination and degradation.^19^ In this study, we identified MARCH3 as a negative regulator of IL-3-triggered signaling. MARCH3 catalyzes K48-linked polyubiquitination of human IL-3Rα at K377 (or murine IL-3Rα at K357). MARCH3-deficiency promotes IL-3-triggered expansion of myeloid cells. In mouse cecal ligation and puncture (CLP) model of sepsis,^20^ MARCH3-deficiency facilitates IL-3-ampilfied polymicrobial sepsis. Our findings suggest that MARCH3 negatively regulates IL-3-triggered physiological functions and inflammatory responses by mediating K48-linked polyubiquitination and degradation of IL-3Rα.

## Results

### MARCH3 negatively regulates IL-3-triggered signaling by downregulating IL-3Rα

**Fig. 1:**
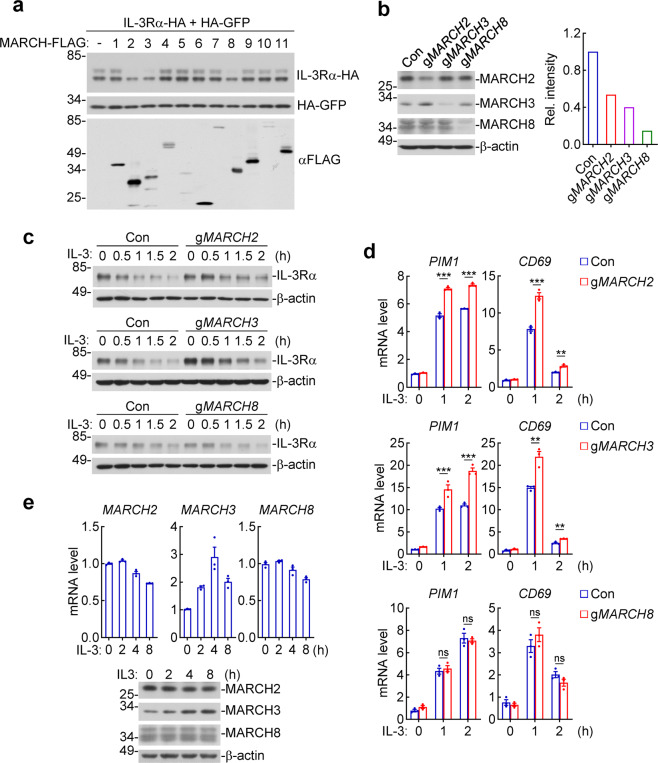
MARCH2 and MARCH3 negatively regulates IL-3-triggered signaling. **a** Effects of overexpression of the MARCH proteins on IL-3Rα level. Human embryonic kidney 293 cells (4 × 10^5^) were transfected with expression plasmids for the indicated FLAG-tagged proteins, HA-tagged IL-3Rα and GFP for 24 h before immunoblots for detection of the indicated proteins. **b** Knockout efficiency of the MARCH proteins. Human erythroleukemia TF-1 cells were transduced with control, g*MARCH2*, g*MARCH3*, or g*MARCH8* by lentiviral-mediated gene transfer. The knockout cells were analyzed by immunoblots for detection of the indicated proteins. **c** Effects of MARCH2, 3, 8-deficiency on IL-3Rα levels. The indicated TF-1 cells (2 × 10^7^) were starved overnight and then stimulated with IL-3 (20 ng/mL) for the indicated times. Cell lysates were immunoprecipitated with anti-IL-3Rα (Santa Cruz Biotechnology, SC-455). The immunoprecipitates were analyzed by immunoblots with anti-IL-3Rα (MYBioSource, MBS2544022). Lysates were analyzed by immunoblots with anti-β-actin. **d** Effects of MARCH2, 3, 8-deficiency on IL-3-induced transcription of downstream genes. The indicated TF-1 cells (5 × 10^5^) were starved overnight and then left untreated or treated with IL-3 (10 ng/mL) for the indicated times before qPCR analysis. Data shown are means ± SEM from one representative experiment performed in triplicate. ** P < 0.01, *** P < 0.001; ns, not significant. **e** Effects of IL-3 on the mRNA and protein levels of MARCH2, 3, 8. TF-1 cells (5 × 10^5^) were starved overnight and then stimulated with IL-3 (20 ng/mL) for the indicated times before qPCR and immunoblotting analysis for detection of the indicated mRNAs and proteins. Data shown are means ± SEM from one representative experiment performed in triplicate. All the experiments were repeated for at least two times with similar results

We have previously demonstrated that the two membrane-associated E3 ubiquitin ligases MARCH3 and MARCH8 negatively regulate IL-1β-triggered inflammation by mediating polyubiquitination of IL-1RI and IL-1RAcP respectively.^18,19^ Therefore, we attempted to investigate whether the level of IL-3Rα is regulated by the MARCH family members. We co-transfected IL-3Rα with each of the 11 MARCH family members in human embryonic kidney 293 cells and examined the levels of IL-3Rα by immunoblots. The results indicated that overexpression of MARCH2, 3 and 8 but not the other examined MARCH proteins markedly downregulated the level of IL-3Rα (Fig. 1a). These data suggest that MARCH2, 3 and 8 function as potential negative regulators of IL-3Rα. In these experiments, the overexpressed FLAG-tagged IL-3Rα was detected as two ~60 and ~70 kDa bands, which were markedly higher than the expected size of ~42 kDa of un-modified IL-3Rα (378 aa). Treatment of overexpressed IL-3Rα by peptide-N-Glycosidase F (PNGase F) reduced their sizes to ~42 kDa in immunoblots (Supplementary Fig. S1a). In addition, endogenous IL-3Rα in human erythroleukemia TF-1 cells that express IL-3Rα,^21^ was detected as a single ~70 kDa band, which was reduced to ~42 kDa following PNGase F treatment (Supplementary Fig. S1b). These results suggest that IL-3Rα is glycosylated in cells.

We then determined the effects of endogenous MARCH2, 3 and 8 on the level of IL-3Rα in TF-1. We generated MARCH2-deficient, 3-deficient and 8-deficient TF-1 cells by CRISPR/Cas9-mediated gene editing (Fig. 1b).^22,23^ Immunoblotting analysis indicated that MARCH3-deficiency markedly increased the level of IL-3Rα before and after IL-3 stimulation comparing to control TF-1 cells. In these experiments, MARCH2-deficiency weakly increased the level of IL-3Rα whereas MARCH8-deficiency had no effects (Fig. 1c). Consistently, MARCH3-deficiency markedly increased IL-3-triggered transcription of downstream *PIM1* and *CD69* genes comparing to control TF-1 cells. MARCH2-deficiency caused weak increases of IL-3-triggered transcription of these downstream genes whereas MARCH8-deficiency had no marked effects (Fig. 1d). These results suggest that MARCH3 negatively regulates the level of IL-3Rα as well as IL-3-triggered signaling, whereas MARCH2 has a weaker role and MARCH8 is not involved in these processes in TF-1 cells. Additionally, we found that MARCH3 but not MARCH2 or MARCH8 was induced by IL-3 at both mRNA and protein levels in TF-1 cells (Fig. 1e), suggesting that MARCH3 is involved in a feed-back negative regulatory mechanism of IL-3-triggered signaling.

**Fig. 2:**
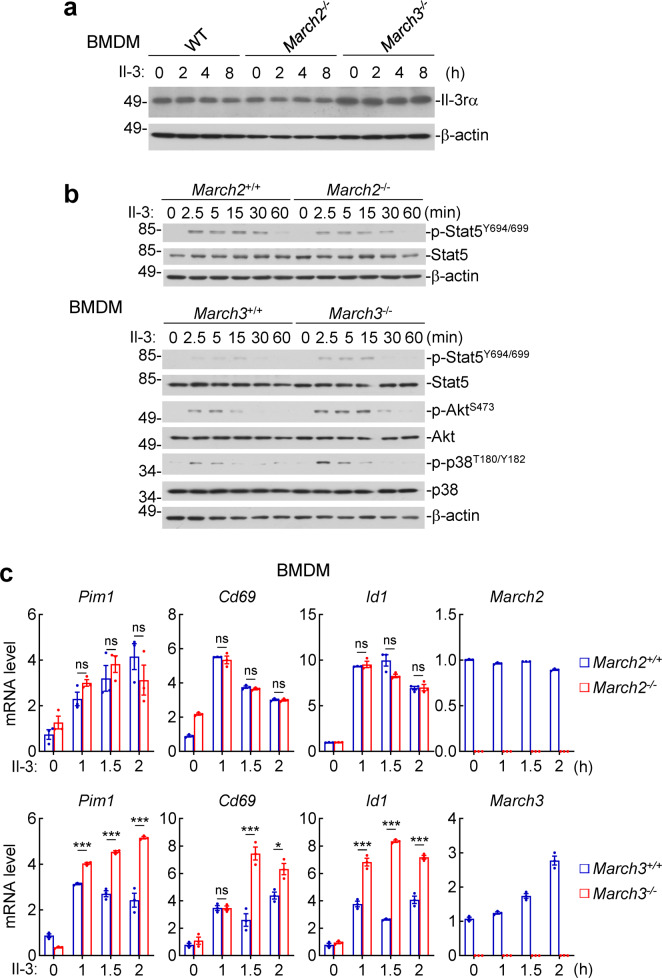
March3-deficiency promotes Il-3-induced signaling in BMDMs. **a** Effects of March2, 3-deficiency on Il-3rα levels. Wild-type, *March2*^*−/−*^ and *March3*^*−/−*^ bone marrow-derived macrophages (BMDMs) (1 × 10^6^) were left untreated or treated with Il-3 (50 ng/mL) for the indicated times before immunoblotting analysis for detection of Il-3rα and β-actin. **b** Effects of March2, 3-deficiency on Il-3-induced phosphorylation of signaling components. The indicated BMDMs (1 × 10^6^) were left untreated or treated with Il-3 (25 ng/mL) for the indicated times before immunoblotting analysis for detection of the indicated proteins. **c** Effects of March2, 3-deficiency on Il-3-induced transcription of downstream genes. Wild-type, *March2*^*−/−*^ or *March3*^*−/−*^ BMDMs (1 × 10^6^) were left untreated or treated with Il-3 (25 ng/mL) for the indicated times before qPCR analysis. Data shown are means ± SEM from one representative experiment performed in triplicate. *P < 0.05, ***P < 0.001; ns, not significant. All the experiments were repeated for at least two times with similar results

To determine whether the functions of MARCH2 and MARCH3 on IL-3-triggered signaling are conserved between human and mouse, we utilized March2-deficient and March3-deficient mice generated by the CRISPR/Cas9 system.^18,24^ In the following text, the designations of homo sapiens genes and proteins are all capitalized, while only the first letter of murine genes and proteins are capitalized. It has been shown that March3-deficiency in mice has no marked effects on development of T and B lymphocytes.^24^ The cellular compositions in the thymus, peripheral lymph nodes and spleen were also comparable between wild-type and *March2*^*−/−*^mice (Supplementary Fig. S2). To determine whether the level of IL-3Rα is regulated by March2 and March3 in primary mouse immune cells, we generated *March2*^*−/−*^ and *March3*^*−/−*^ bone marrow-derived macrophages (BMDMs). Immunoblotting analysis indicated that deficiency of March3 but not March2 up-regulated the level of endogenous Il-3rα before and after Il-3 stimulation (Fig. 2a). March3-deficiency increased Il-3-triggered phosphorylation of Akt^S473^, Stat5^Y694/699^, and p38^T180/Y182^, which are hallmarks of activation of Il-3-triggered signaling pathways.^12^ In these experiments, March2-deficiency had no marked effects on phosphorylation of Stat5^Y694/699^ (Fig. 2b). Consistently, qPCR analysis indicated that March3- but not March2-deficiency markedly increased Il-3-induced transcription of *Pim1*, *Cd69*, and *Id1* genes in BMDMs (Fig. 2c). These results suggest that March3 but not March2 specifically down-regulates Il-3rα level as well as negatively regulates Il-3-triggered signaling in mouse BMDMs.

### MARCH3 mediates K48-linked polyubiquitination of IL-3Rα at K377

**Fig. 3:**
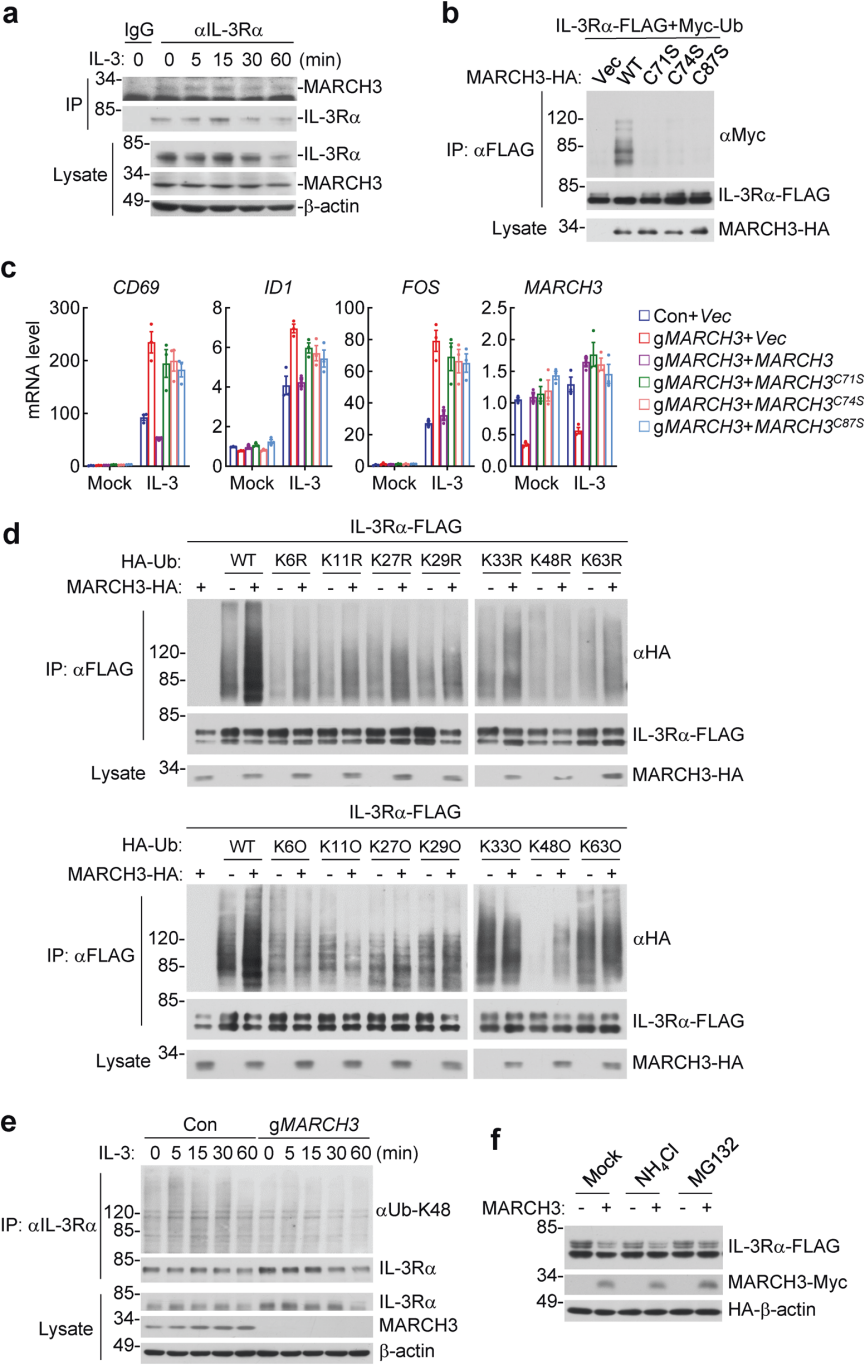
MARCH3 mediates K48-linked polyubiquitination of IL-3Rα. **a** Endogenous MARCH3 is associated with IL-3Rα. Human erythroleukemia TF-1 cells (2 × 10^7^) were starved overnight and then left untreated or treated with IL-3 (20 ng/mL) for the indicated times. Cell lysates were immunoprecipitated with control mouse IgG (IgG) or anti-IL-3Rα as indicated. The immunoprecipitates were analyzed by immunoblots with rabbit anti-IL-3Rα and mouse anti-MARCH3 as indicated. Lysates were analyzed by immunoblots with rabbit anti-IL-3Rα, mouse anti-MARCH3 and anti-β-actin to determine levels of the respective endogenous proteins. **b** MARCH3 but not its E3 ligase-inactive mutants mediates polyubiquitination of IL-3Rα. Human embryonic kidney 293 cells (2 × 10^6^) were transfected with expression plasmids for FLAG-tagged IL-3Rα, Myc-tagged wild-type ubiquitin (Ub) and the indicated HA-tagged wild-type or mutant MARCH3 for 20 h. Cell lysates were immunoprecipitated with anti-FLAG. The immunoprecipitates were denatured and then re-immunoprecipitated with anti-FLAG. The final immunoprecipitates and cell lysates were analyzed by immunoblots for detection of the indicated proteins. **c** MARCH3-deficient TF-1 cells were reconstituted with wild-type MARCH3 and its E3 ligase-inactive mutants by lentiviral-mediated gene transfer. The reconstituted cells (5 × 10^5^) were starved overnight and then stimulated with IL-3 (10 ng/mL) for 1 h before qPCR analysis for mRNA levels of the indicated genes. Data shown are means ± SEM from one representative experiment performed in triplicate. **d** Linkage types of polyubiquitination of IL-3Rα. HEK293 cells (2 × 10^6^) were transfected with expression plasmids for FLAG-tagged IL-3Rα, HA-tagged MARCH3 and HA-tagged wild-type ubiquitin (Ub) or its mutants for 20 h. Cell lysates were immunoprecipitated with anti-FLAG. The immunoprecipitates were denatured and then re-immunoprecipitated with anti-FLAG. The final immunoprecipitates and cell lysates were analyzed by immunoblots for detection of the indicated proteins. **e** Effects of MARCH3-deficiency on IL-3-induced K48-linked polyubiquitination of IL-3Rα. Control and MARCH3-deficient TF-1 (2 × 10^7^) cells were starved overnight and then left untreated or stimulated with IL-3 (20 ng/mL) for the indicated times. Cell lysates were then immunoprecipitated with anti-IL-3Rα. The immunoprecipitates were denatured and then re-immunoprecipitated with anti-IL-3Rα. The final immunoprecipitates were analyzed by immunoblots with anti-Ub-K48 or anti-IL-3Rα. Cell lysates were analyzed by immunoblots for detection of the indicated proteins. **f** Effects of inhibitors on MARCH3-mediated degradation of IL-3Rα. HEK293 cells (4 × 10^5^) were transfected with expression plasmids for FLAG-tagged IL-3Rα, Myc-tagged MARCH3 and HA-tagged β-actin as indicated for 12 h, and then treated with the lysosomal inhibitor NH_4_Cl (5 mM) or the proteasome inhibitor MG132 (0.5 μM) for 6 h before immunoblotting analysis with anti-FLAG, anti-Myc and anti-HA respectively. All the experiments were repeated twice with similar results

To investigate the molecular mechanisms on how MARCH3 downregulates IL-3Rα, we firstly examined whether MARCH3 is associated with IL-3Rα. Endogenous coimmunoprecipitation experiments showed that MARCH3 was constitutively associated with IL-3Rα in TF-1 cells (Fig. 3a). Since MARCH3 is an E3 ubiquitin ligase and mediates downregulation of IL-3Rα, we determined whether MARCH3 mediates polyubiquitination of IL-3Rα. In mammalian overexpression system, wild-type MARCH3, but not its ligase-inactive mutants, including C71S, C74S, and C87S, markedly enhanced polyubiquitination of IL-3Rα (Fig. 3b). Furthermore, reconstitution of MARCH3 but not MARCH3^C71S^, MARCH3^C74S^ or MARCH3^C87S^ into MARCH3-deficiency TF-1 cells inhibited IL-3-induced transcription of *CD69, ID1* and *FOS* genes (Fig. 3c). These results suggest that MARCH3 mediates polyubiquitination and degradation of IL-3Rα.

We next determined the types of polyubiquitination of IL-3Rα mediated by MARCH3. By co-transfection of IL-3Rα with ubiquitin mutants in which only one lysine reside is mutated to arginine (K/R), we found that MARCH3 failed to enhance polyubiquitination of IL-3Rα only when K48 was mutated to arginine. By co-transfection of IL-3Rα with ubiquitin mutants that contain only a single lysine residue (K/O), we found that MARCH3 markedly increased K48-linked but not other lysine-linked polyubiquitination of IL-3Rα (Fig. 3d). Most importantly, MARCH3-deficiency inhibited IL-3-induced K48-linked polyubiquitination of IL-3Rα (Fig. 3e). MARCH3-mediated downregulation of glycosylated IL-3Rα was rescued by the proteasome inhibitor MG132 but not the lysosomal inhibitor NH_4_Cl (Fig. 3f). These results suggest that MARCH3 catalyzes K48-linked polyubiquitination of IL-3Rα and promotes its proteasomal degradation following IL-3 stimulation.

**Fig. 4:**
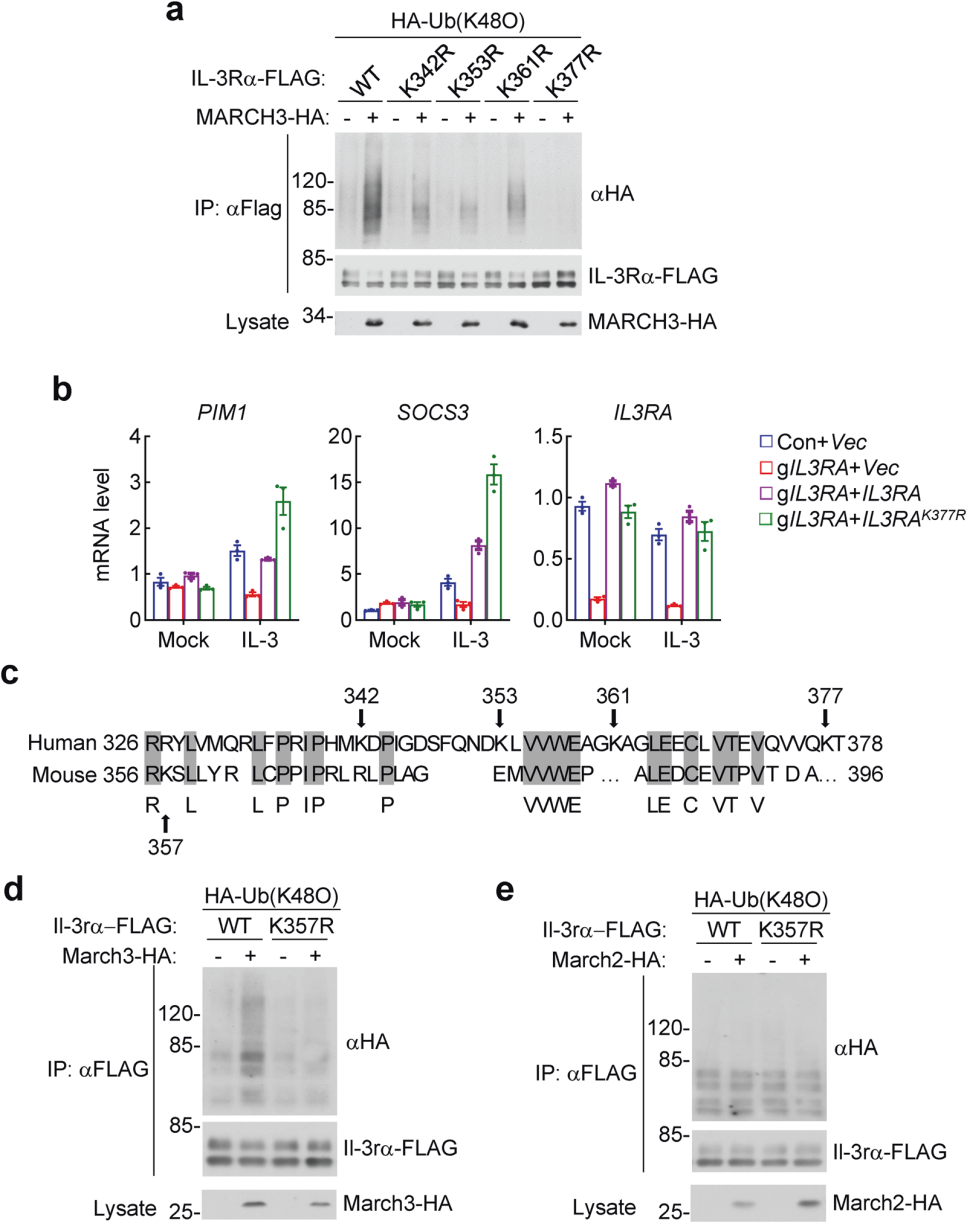
MARCH3 mediates K48-linked polyubiquitination of IL-3Rα at K377. **a** MARCH3 catalyzes K48-linked polyubiquitination of IL-3Rα at K377. Human embryonic kidney 293 cells (2 × 10^6^) were transfected with the indicated plasmids for 20 h. Cell lysates were then immunoprecipitated with anti-FLAG. The immunoprecipitates were denatured and then re-immunoprecipitated with anti-FLAG. The final immunoprecipitates and cell lysates were analyzed by immunoblots with anti-HA or anti-FLAG. HA-Ub (K48O), HA-tagged ubiquitin with all lysine residues mutated to arginine except K48. **b** IL-3Rα^K377R^ has increased ability than IL-3Rα in mediating IL-3-triggered signaling. IL-3Rα-deficient human erythroleukemia TF-1 cells were reconstituted with wild-type IL-3Rα and IL-3Rα^K377R^ by lentiviral-mediated gene transfer. The reconstituted TF-1 cells (5 × 10^5^) were starved overnight and stimulated with IL-3 (10 ng/mL) for 1 h before qPCR analysis for mRNA levels of the indicated genes. Data shown are means ± SEM from one representative experiment performed in triplicate. **c** Sequence alignment of the cytoplasmic domains of human and murine IL-3Rα. The sequences are corresponding to aa326-378 of human IL-3Rα and aa356-396 of murine Il-3rα. **d** March3 but not March2 catalyzes K48-linked polyubiquitination of Il-3rα at K357. HEK293 cells (2 × 10^6^) were transfected with the indicated plasmids for 20 h. Cell lysates were then immunoprecipitated with anti-FLAG. The immunoprecipitates were denatured and then re-immunoprecipitated with anti-FLAG. The final immunoprecipitates and cell lysates were analyzed by immunoblots with anti-FLAG or anti-HA. All the experiments were repeated for at least two times with similar results

We next mapped the residues in IL-3Rα that are polyubiquitinated by human MARCH3. There are four lysine residues in the cytoplasmic domain (aa326–378) of IL-3Rα. We individually mutated each of these lysine residues to arginine and examined whether these IL-3Rα mutants could be modified by MARCH3-mediated K48-linked polyubiquitination. The results indicated that MARCH3 catalyzed K48-linked polyubiquitination of IL-3Rα^K342R^, IL-3Rα^K353R^, and IL-3Rα^K361R^ but not IL-3Rα^K377R^ (Fig. 4a). Reconstitution of IL-3Rα^K377R^ in IL-3Rα-deficient cells increased IL-3-induced transcription of downstream genes in comparison to cells reconstituted with wild-type IL-3Rα (Fig. 4b). These results suggest that human MARCH3 catalyzes K48-linked polyubiquitination of IL-3Rα at K377, which promotes its proteasomal degradation and inhibition of IL-3-trigggered signaling.

Sequence alignment indicates that the lysine residues in the cytoplasmic domain of human IL-3Rα are not conserved in mouse. There is only one lysine residue, K357, in the cytoplasmic domain of murine Il-3rα (Fig. 4c). We mutated this lysine residue to arginine and examined whether the Il-3rα mutant could be modified with K48-linked polyubiquitination by March3. We found that murine March3 catalyzed K48-linked polyubiquitination of wild-type Il-3rα but not Il-3rα^K357R^ (Fig. 4d), whereas murine March2 failed to mediate K48-linked polyubiquitination of both Il-3rα and Il-3rα^K357R^ (Fig. 4e). These results suggest that murine March3 but not March2 mediates K48-linked polyubiquitination of murine Il-3rα at K357.

### March3-deficiency promotes Il-3-triggered expansion of myeloid cells and sepsis

**Fig. 5:**
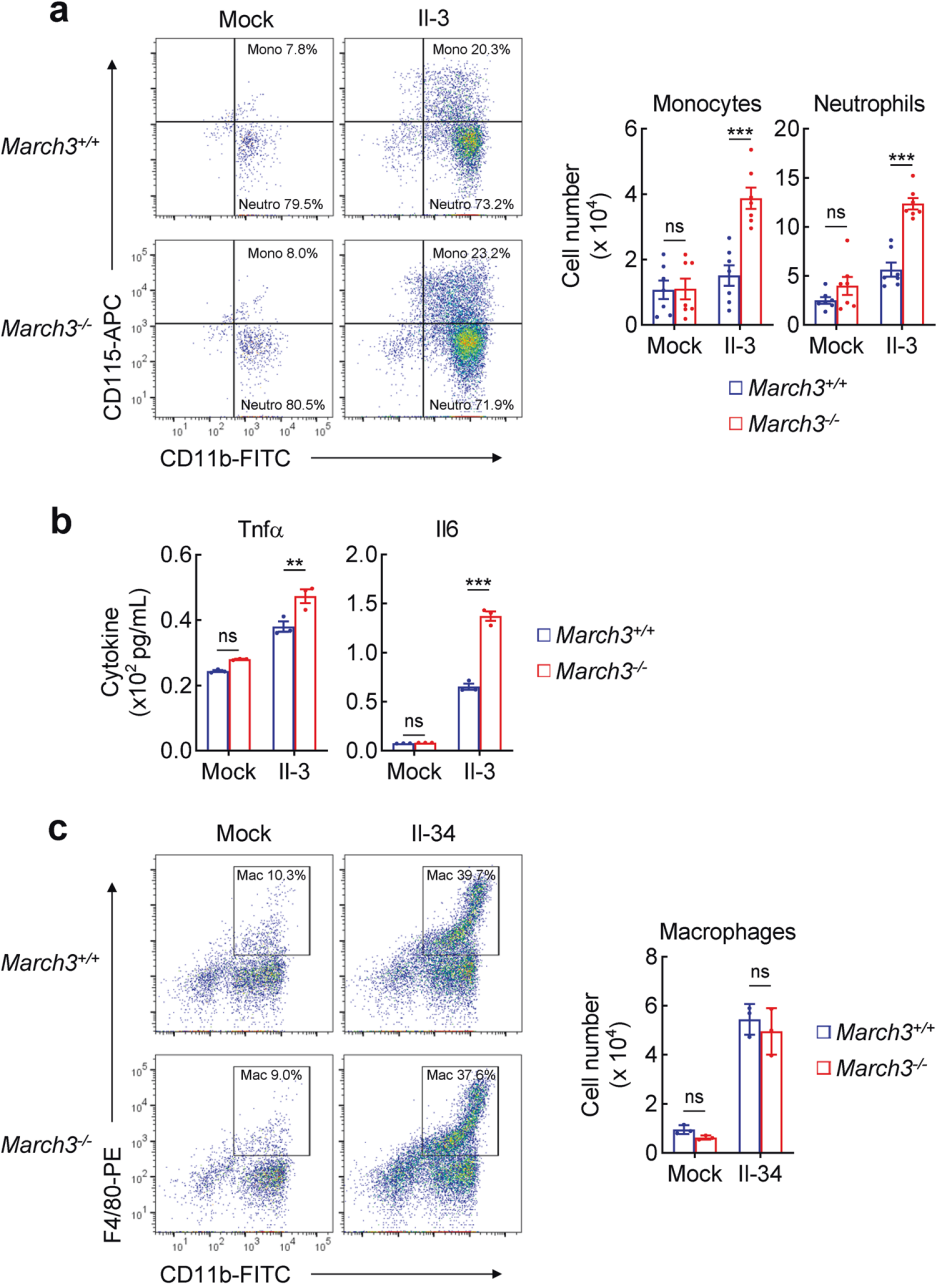
March3-deficiency promotes Il-3-triggered expansion of myeloid cells. **a** *March3*^*+/+*^ or *March3*^*−/−*^ Lin^−^ bone marrow cells were treated with Il-3 (5 ng/mL) for 4 days before flow cytometry analysis (left panel) and counting of cell numbers (right histograph). Data shown in the right histographs are mean ± SEM, *n* = 7. ***P < 0.001; ns not significant. **b** *March3*^*+/+*^ or *March3*^*−/−*^ Lin^−^ bone marrow cells were treated with Il-3 (5 ng/mL) for 4 days. The culture media of the cells were collected for measurement of Tnf-α and Il-6 levels by ELISA. Data shown are mean ± SEM, *n* = 3. **P < 0.01, ***P < 0.001; ns not significant. **c** The indicated Lin^-^ bone marrow cells were treated with Il-34 (100 ng/mL) for 4 days before flow cytometry analysis (left panel) and counting of cell numbers (right histograph). Data shown in the right histograph are mean ± SEM, *n* = 3. ns not significant

It has been previously demonstrated that IL-3 promotes expansion of myeloid cells. We prepared wild-type and *March3*^*−/−*^ Lin^−^ bone marrow cells, which contain predominantly hematopoietic stem and progenitor cells. We found that after Il-3 stimulation for 4 days, the number of *March3*^*−/−*^ monocytes (CD11b^+^CD115^+^) and neutrophils (CD11b^+^CD115^−^) was 2–3 folds to that of wild-type cells (Fig. 5a). In addition, March3-deficiency markedly increased Il-3-induced production of Tnf-α and Il-6 in these cells (Fig. 5b), which are primary inflammatory cytokines produced during septic shock.^25^ IL-34 is shown to support the survival of monocytes and promote the formation of macrophage colonies in bone marrow cell cultures.^26–28^ In similar experiments, Il-34-induced expansion of macrophages (CD11b^+^F4/80^+^) was comparable between wild-type and *March3*^*−/−*^ bone marrow cells (Fig. 5c). These results suggest that March3 specifically regulates Il-3-induced expansion of myeloid cells and cytokine production.

**Fig. 6:**
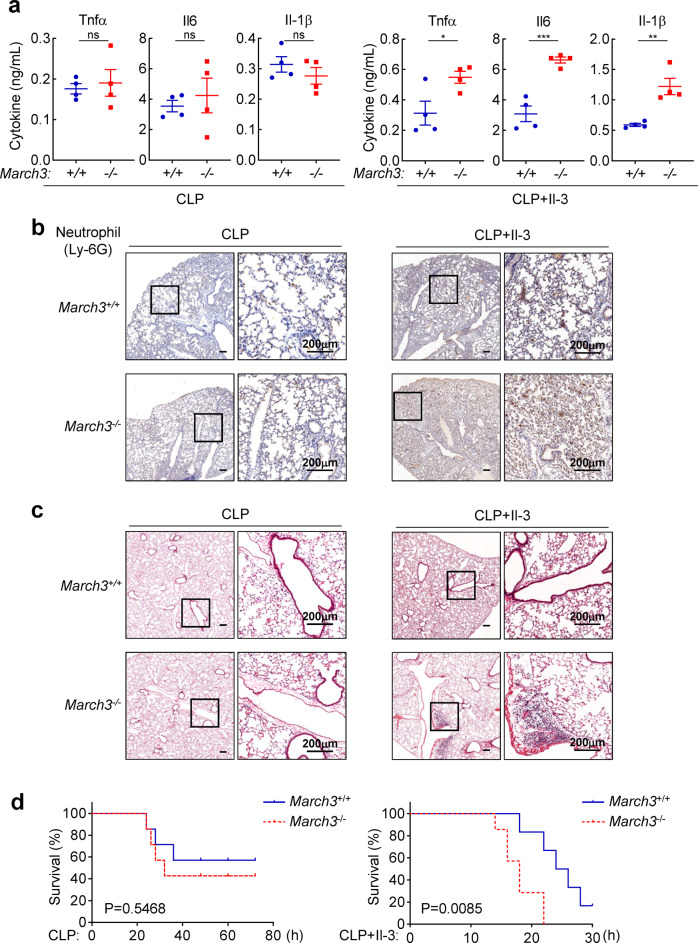
March3-deficiency promotes Il-3-amplified sepsis in mice. **a** Effects of March3-deficiency on serum levels of Tnf-α, Il-6 and Il-1β. Sex-matched and age-matched *March3*^*+/+*^ and *March3*^*−/−*^ mice were subjected to CLP and then injected via tail vein with Il-3 (3 μg each mouse) or PBS for 4 h before measurement of serum cytokines by ELISA. Results are represented as mean ± SEM, *n* = 4. *P < 0.05, **P < 0.01, ***P < 0.001; ns not significant. **b**, **c** IHC and H&E staining of lung tissues. The mice were subjected to CLP and injected twice with Il-3 (3 μg each mouse) or PBS via tail vein at 30 min and 12 h after CLP. Mice were sacrificed at 24 h after CLP for IHC staining with Ly-6G (**b**) or H&E staining (**c**). **d** Effects of March3-deficiency on CLP-induced death of mice. For the left graph, *March3*^*+/+*^ and *March3*^*−/−*^ mice (*n* = 7 for each group) were subjected to CLP, and the survival of mice was monitored every 2 h. For the right graph, *March3*^*+/+*^ (*n* = 6) and *March3*^*−/−*^ (*n* = 7) mice were subjected to CLP and injected twice with Il-3 (3 μg each mouse) via tail vein at 30 min and 12 h after CLP. The survival of mice was monitored every 2 h. The survival curves were generated by Kaplan–Meier methods followed by log-rank test analysis

It has been shown that Il-3 promotes inflammation and cytokine storms in murine sepsis models.^4^ To investigate the roles of March3 in sepsis, sex-matched and age-matched *March3*^*+/+*^ and *March3*^*−/−*^ mice were subjected to cecal ligation and puncture (CLP) procedures, which have been used as a model for polymicrobial sepsis.^20^ We found that *March3*^*−/−*^ mice treated with CLP and Il-3 produced higher levels of serum Tnf-α, Il-6, and Il-1β than wild-type mice. In contrast, the serum cytokine levels in *March3*^*+/+*^ and *March3*^*−/−*^ mice treated with CLP alone were comparable (Fig. 6a). After CLP and Il-3 administration, *March3*^*−/−*^ mice had more Ly-6C^high^ monocytes and neutrophils in the bone marrow, peripheral blood, and lung as suggested by flow cytometry analysis (Supplementary Fig. S3a–c). Immunohistochemistry also indicated that *March3*^*−/−*^ mice accumulated markedly more neutrophils (Ly-6G^+^) in the lung after CLP and Il-3 administration comparing to the wild-type mice (Fig. 6b). Hematoxylin-eosin (H&E) staining indicated that more inflammatory cell infiltration and aggregation were observed in the lungs of *March3*^*−/−*^ mice than their wild-type counterparts after CLP and Il-3 administration (Fig. 6c). *March3*^*−/−*^ mice were more susceptible to septic death than the wild-type mice following CLP and Il-3 administration. In similar experiments, *March3*^*−/−*^ and *March3*^*+/+*^ mice had no significant difference in septic death induced by CLP alone (Fig. 6d). These results suggest that March3 attenuates Il-3-amplified inflammation in sepsis.

## Discussion

The IL-3/IL-3Rα axis is involved in hematopoiesis, sepsis and several inflammatory disorders, therefore, may serve as targets for development of drugs for the related diseases. In this study, we identified MARCH3 as a negative regulator of IL-3-triggered effects by targeting IL-3Rα for K48-linked polyubiquitination and proteasomal degradation.

Our results indicated that MARCH3 was constitutively associated with IL-3Rα. Overexpression of MARCH3 downregulated the level of IL-3Rα, whereas MARCH3-deficiency upregulated the level of endogenous IL-3Rα in unstimulated as well as IL-3-stimulated cells. Overexpression of MARCH3 mediated K48-linked polyubiquitination and proteasomal degradation of IL-3Rα, whereas MARCH3-deficiency increased K48-linked polyubiquitination and protein level of IL-3Rα in unstimulated and IL-3-stimulated cells. These results suggest that MARCH3 constitutively regulates IL-3Rα level in the cell. Interestingly, MARCH3 is upregulated by IL-3 stimulation, suggesting that MARCH3 acts as a feed-back negative regulator of IL-3-triggered signaling and biological effects.

Our results suggest that regulation of IL-3Rα by MARCH3 is conserved between human and mouse. However, our results also suggest that human and mouse IL-3Rα are differentially regulated by the MARCH proteins. While both human MARCH2 and MARCH3 are involved in regulation of IL-3Rα, only murine March3 but not March2 regulates Il-3rα level. Sequence alignment of human and murine IL-3Rα suggests that the lysine residues in the cytoplasmic domains of these receptors are not conserved. Human MARCH3 catalyzed K48-linked polyubiquitination of IL-3Rα at K377, whereas mouse March3 catalyzed K48-linked polyubiquitination of Il-3rα at K357. Consistently, mouse March2 did not catalyze K48-linked polyubiquitination of Il-3rα. These results suggest that human and mouse IL-3Rα is differentially regulated by MARCH2 and MARCH3. Recently, one report suggests that the murine E3 ubiquitin ligase RNFT2 causes polyubiquitination and proteasomal degradation of IL-3Rα in murine overexpression system.^29^ It is unknown whether RNFT2 mediates K48-linked polyubiquitination of IL-3Rα in un-transfected cells. In addition, whether RNFT2 regulates the classical IL-3Rα-mediated signaling events such as the JAK2-STAT5 pathway is unknown.

Consistent with a role of MARCH3 in regulating IL-3Rα level, MARCH3-defieincy enhanced IL-3-triggered signaling and transcription of downstream effector genes in human TF-1 cells. March3-deficiency upregulated the level of endogenous Il-3rα and potentiated Il-3-induced signaling in BMDMs. Biologically, March3-deficiency promoted Il-3-triggered expansion of myeloid cells as well as production of inflammatory cytokines. In the CLP sepsis model, March3-deficiency increased Il-3-induced serum cytokine levels, and promoted Il-3-amplified inflammatory response and sepsis.

Based on our results, we conclude that MARCH3 plays an important role in regulating IL-3-triggered physiological functions and pathological processes. These findings contribute to our understanding of the delicate regulatory mechanisms of IL-3Rα mediated signaling as well as development of therapeutic intervention for related inflammatory diseases. Previously, it has been reported that MARCH3 downregulated IL-1RI but not its co-receptor IL-1RAcP.^18^ This observation and the findings from our current study together suggest that MARCH3 versatilely regulates inflammation response by targeting distinct components of the IL receptor family.

## Materials and methods

### Reagents, antibodies, and cells

Recombinant human GM-CSF (PeproTech, 300-03) and IL-3 (PeproTech, 200-03), murine Il-3 (PeproTech, 213-13) and Il-34 (Sino Biological, 50055-M08H), M-MLV reverse transcriptase (Invitrogen, 28025-013); RiboLock RNase inhibitor (Thermo Scientific, EO0382), RNAiso plus (Takara Bio, 9109), SYBR Green mix (Bio-Rad, 172-5274); MG132 (MCE, HY-13259), polybrene (Millipore, TR-1003-G); RPMI 1640 medium (Gibco, C11875500BT), Dulbecco’s Modified Eagle Medium (Gibco, C11995500BT), FBS (Cellmax, SA211.02), penicillin/streptomycin (Gibo, 15140-122); ELISA kits for murine Tnf-α (BioLegend, 430904), Il-6 (BioLegend, 431304), Il-1β (Boster bio, EK0394); PNGase F (NEW ENGLAND BioLabs, P0704S); Mouse monoclonal antibodies against β-actin (Sigma-Aldrich, A2228), FLAG^®^ M2 (Sigma-Aldrich, F3165), HA (BioLegend, 901515), Myc (Cell Signaling Technology, 2276), phosphor-p38^T180/Y182^ (Cell Signaling Technology, 9216), human IL-3Rα (for Co-IP) (Santa Cruz Biotechnology, SC-455); Rabbit antibodies against phospho-STAT5^Y694/699^ (Cell Signaling Technology, 4322), phospho-AKT^S473^ (Cell Signaling Technology, 4060), p38 (Cell Signaling Technology, 8690), STAT5 (Cell Signaling Technology, 94205), AKT (Cell Signaling Technology, 4691), human IL-3Rα (for immunoblot) (MYBioSource, MBS2544022), murine Il-3rα (MYBioSource, MBS2542745), murine March2 (Abcam, ab220292), MARCH8 (Proteintech, 14119-1-AP), K48-linkage specific polyubiquitin (Abcam, ab140601); HRP-conjugated mouse anti-HA (Sigma-Aldrich, H6533); Goat anti-mouse IgG (Jackson Immuno Research, 115-035-003) were purchased from the indicated companies. Mouse antisera against MARCH3 were raised against recombinant human MARCH3 (2–144aa), and rabbit antisera against human MARCH2 were raised against recombinant human MARCH2 (1–137aa). Human embryonic kidney 293 and human erythroleukemia TF-1 cells were obtained from ATCC.

### Constructs

Expression plasmids for FLAG-tagged, HA-tagged, or Myc-tagged MARCH1-11 and their mutants, IL-3Rα and its mutants, and Il-3rα and its mutant were constructed by standard molecular biology techniques. Guide-RNA plasmids targeting *MARCH2, MARCH3*, *MARCH8*, and *IL3RA* were constructed into lentiCRISPR v2 vector.

### Mice

All animal experiments were performed in accordance with the Wuhan University Animal Care and Use Committee guidelines. *March2*^*−/−*^ and *March3*^*−/−*^ mice were generated as previously described.^18,24^ The mice were bred in a specific pathogen-free facility at Wuhan University Medical Research Institute. Six to eight-week-old mice were used in the experiments and littermates were used as controls.

### Mouse immune cell experiments

For preparation of BMDMs, mouse bone marrow-derived monocytes (1 × 10^7^) were cultured in 100 mm dishes in 5 mL 10% M-CSF-containing conditional medium from L929 cells for 3–6 days.

For the myeloid cell proliferation experiments, mouse bone marrow cells were lysed with RBC to remove red blood cells. Lineage negative cells (Lin^−^) were enriched with hematopoietic progenitor cell enrichment kit (STEMCELL, 19856A). The cells were seeded at a density of 1 × 10^5^/mL in 12-well flat-bottom dishes and treated with recombinant Il-3 (5 ng/mL) or Il-34 (100 ng/mL) for the indicated times before flow cytometry.

### Coimmunoprecipitation and immunoblot analysis

For transient transfection and coimmunoprecipitation experiments, HEK293 cells (2 × 10^6^) were transfected with the indicated plasmids by a standard calcium phosphate precipitation method. The cells were then lysed in 1 mL NP-40 lysis buffer (20 mM Tris-HCl, pH 7.5, 150 mM NaCl, 1 mM EDTA, 1% NP-40, 10 μg/mL aprotinin, 10 μg/mL leupeptin and 1 mM phenylmethylsulfonyl fluoride). The lysates were centrifuged at 12,000×*g* for 10 min at 4 °C. For each immunoprecipitation, the supernatant was incubated with 0.5 μg of the indicated antibody and 35 μL of 50% slurry of GammaBind G Plus-Sepharose (GE healthcare, 17-0618-05) at 4 °C for 3 h. The beads were washed for three times with 1 mL lysis buffer containing 500 mM NaCl. The bead-associated proteins were separated by SDS-PAGE, followed by immunoblotting analysis with the indicated antibodies. For endogenous coimmunoprecipitation experiments, the cells were starved overnight in medium without FBS and stimulated with IL-3 for the indicated times or left untreated before coimmunoprecipitation and immunoblotting analysis.

### Ubiquitination assays

Immunoprecipitates were re-extracted in lysis buffer containing 1% SDS and denatured by heating for 10 min. The samples were centrifuged at 12,000×*g* for 10 min, and the supernatants were diluted with NP-40 lysis buffer until the concentration of SDS was decreased to 0.1%, followed by re-immunoprecipitation with the indicated antibodies. Ubiquitin-modified proteins were detected by immunoblots with indicated antibodies.

### qPCR

Total RNA was isolated for qPCR analysis to measure mRNA levels of the indicated genes according to the manufacturer’s protocol (TaKaRa). Data shown are relative abundance of the indicated mRNA normalized to that of human *GAPDH or mouse Gapdh* gene. Sequences of gene-specific primer pairs are shown below.

Human *GAPDH*: GACAAGCTTCCCGTTCTCAG (forward) and GAGTCAACGGATTTGGTCGT (reverse);

Human *SOCS3*: CATCTCTGTCGGAAGACCGTCA (forward) and GCATCGTACTGGTCCAGGAACT (reverse);

Human *FOS*: GCCTCTCTTACTACCACTCACC (forward) and AGATGGCAGTGACCGTGGGAAT (reverse);

Human *PIM1*: TCTACTCAGGCATCCGCGTCTC (forward) and CTTCAGCAGGACCACTTCCATG (reverse);

Human *CD69*: GCTGGACTTCAGCCCAAAATGC (forward) and AGTCCAACCCAGTGTTCCTCTC (reverse);

Human *ID1*: GTTGGAGCTGAACTCGGAATCC (forward) and ACACAAGATGCGATCGTCCGCA (reverse);

Human *MARCH2*: CTAACACCAGCTACTGCGAGCT (forward) and GGAAACACACCATGTCGCAGCA (reverse);

Human *MARCH3*: CTGGCTGTCATCCTCAAACACC (forward) and TGTCGCCAAACAGAGTCCGCTT (reverse);

Human *MARCH8*: CTCTCGCACTTCTATCACGCCA (forward) and AAGTGGAGGCTTCCTGTGCAGT (reverse);

Mouse *Gapdh*: ACGGCCGCATCTTCTTGTGCA (forward) and ACGGCCAAATCCGTTCACACC (reverse);

Mouse *Pim1*: TGTCTCTTCAGAGTGTCAGC (forward) and CGGATTTCTTCAAAGGAGGG (reverse);

Mouse *Cd69*: TCTCATTGCCTTAAATGTGGG (forward) and GTAGCAACATGGTGGTCAG (reverse);

Mouse *Id1*: AACTCGGAGTCTGAAGTCG (forward) and GACACAAGATGCGATCGTC (reverse);

Mouse *March2*: ATTCACAGAGTGACTGTCCCTT (forward) and GACAGCCATTTCTCCAGGCAGC (reverse);

Mouse *March3*: GGAAGCAGCCAAGAGGACTT (forward) and TGCAAACCTGAAGTGGCAGA (reverse).

### ELISA

Eight-week old *March3*^*+/+*^ and *March3*^*−/−*^ mice were subjected to CLP and injected i.v. with Il-3 for 4 h. The sera of mice were collected for measurement of Tnf-α, Il-6, and Il-1β by ELISA according to the manufacturer’s protocol.

### Retroviral-mediated gene transfer

HEK293 cells plated on 100 mm dishes were transfected with pMSCV-PuroR (10 μg) together with the pGag-Pol (10 μg) and pVSV-G (3 μg) plasmids. Two days after transfection the viruses were harvested to infect TF-1 cells in the presence of polybrene (8 μg/mL). One day later, the infected cells were selected with puromycin (2 μg/mL) for at least 5 days.

### CRISPR-Cas9 knockout

The protocols for gene engineering using the CRISPR-Cas9 system were previously described.^22,23^ Briefly, double-stranded oligonucleotides corresponding to the target sequences were cloned into the lentiCRISPR V2 plasmid, which was transfected with packaging plasmids psPAX2 and pMD2.G into HEK293 cells. Two days after transfection, the viruses were harvested to infect TF-1 cells in the presence of polybrene (8 μg/mL). One day later, the infected cells were selected with puromycin (2 μg/mL) for at least 5 days. The following oligonucleotides were used to construct the respective gRNA plasmids.

Control: 5′-GTAGTCGGTACGTGACTCGT-3′

g*IL3RA*: 5′-CAGGTCGTACTGGACGTCCG-3′;

g*MARCH2* #1: 5′-GCCCGTAGCCTCCACGACCT-3′;

g*MARCH2* #2: 5′-CCACATACTGGGGCGGTCCG-3′;

g*MARCH3* #1: 5′-TGCACCCGTGGTGAAGACGG-3′;

g*MARCH3* #2: 5′- GATTGTGGCAGCCTAGTGAA-3′;

g*MARCH8*: 5′-CGTGATAGAAGTGCGAGAGA-3′.

### Flow cytometry

For analysis of cellular compositions in thymus, peripheral lymph nodes and spleen in *March2*^*−/−*^ and *March3*^*−/−*^ mice, the tissues were collected and single-cell suspensions were prepared. After depletion of red blood cells by ammonium-chloride-potassium (ACK) lysing buffer, cells were subjected to stain with the indicated antibodies for 30 min followed by flow cytometry analysis.

For analysis of myeloid cell expansion following Il-3 and Il-34 stimulation, cells were subjected to stain with the indicated antibodies for 30 min and analyzed by flow cytometry.

The following antibodies were used for flow cytometric analyses: anti-CD115-APC (eBioscience, 17-1152-82); anti-CD11b-FITC (BioLegend, 101206); anti-F4/80-PE (eBioscience, 12-4801-82); anti-CD3-FITC (BD Biosciences, 561798); anti-CD4-PE (BD Biosciences, 553048); anti-CD4-APC (BD Biosciences, 553051); anti-CD8-PB (BD Biosciences, 558106); anti-B220-APC (BD Biosciences, 553092); anti-F4/80-APC (BioLegend, 123115); anti-Ly6C-PE/Cy7 (BioLegend, 128017). Data were acquired on a Fortessa™ X-20 (BD Biosciences) and analyzed with FlowJo software.

### Animal models

The cecal ligation and puncture (CLP) model of sepsis was carried out as previously described.^20^ The peritoneal cavity was opened during anesthesia (0.1% pentobarbital, 7 μL/g). For survival experiments, ~60–80% of the cecum was ligated to induce high-grade CLP; for other analysis, ~30–50% of the cecum was ligated to induce mid-grade CLP. The distal end of the cecum was then perforated using a 19G needle. The mice were injected via the tail vein with recombinant Il-3 (3 μg in 150 μL PBS for each mouse) twice at 30 min and 12 h after CLP.

For histology analysis, the lungs from mice were harvested 1 day after CLP and embedded in 4% PFA. The lungs were filled with 4% PFA through the tracheas prior to harvesting. Serial 6 µm thick fresh-frozen sections were prepared and stained with hematoxylin and eosin (H&E) for overall histological analysis. For immunohistochemical staining, the sections were incubated with anti-Ly-6G (1A8, BioLegend) following the manufacturer’s protocol.

For preparation of mouse lung single cell suspensions, the lung was cut into small pieces and transferred into a gentleMACS C Tube (Miltenyi Biotec, 130-093-237) with the enzyme mix containing 2.35 mL of 1640, 100 μL of Enzyme D, 50 μL of Enzyme R and 12.5 μL of Enzyme A from a Tumor Dissociation Kit (Miltenyi Biotec, 130-096-730). The C Tube was tightly closed and attached onto the sleeve of the gentleMACS^TM^ Octo Dissociator (Miltenyi Biotech, 130-095-937) with the tumor isolation program. After termination of the program, the C Tube was detached from the Dissociator and incubated at 37 °C for 45 min. The tumor isolation program were repeated twice and the suspension samples were filtered through MACS SmartStrainers (70 μm, Miltenyi Biotec, 130-110-916) and processed for flow cytometry.

### Statistics

Data were analyzed using Student’s unpaired *t*-test with GraphPad Prism 8 and Microsoft Excel. For the mouse survival study, Kaplan–Meier survival curves were generated and analyzed by Log-Rank test. The number of asterisks represents the degree of significance with respect to *P* values, with the latter presented within each figure or figure legend.

## Supplementary information

Supplementary Figures 1–3
