# Supplementary material for: MARCH3 negatively regulates IL-3-triggered inflammatory response by mediating K48-linked polyubiquitination and degradation of IL-3Rα
Source: Signal Transduct Target Ther. 2022 Jan 24;7:21. doi: 10.1038/s41392-021-00834-7 (PMC8786845; doi:10.1038/s41392-021-00834-7)
Supplement: Supplementary file 1 — Supplementary Figures 1–3 [file 41392_2021_834_MOESM1_ESM.pdf]

Supplementary Materials for

MARCH3 negatively regulates IL-3-triggered inflammatory response by mediating K48-linked polyubiquitination and degradation of IL-3Rα

Lu Feng, Chen Li, Lin-Wen Zeng, Deng Gao, Yu-Hao Sun, Li Zhong, Heng Lin, Hong-Bing Shu*, Shu Li*

*Corresponding to:

Dr. Hong-Bing Shu
Email: shuh@whu.edu.cn

Dr. Shu Li
Email: shuli@whu.edu.cn

This PDF file includes:

Figures S1 to S3

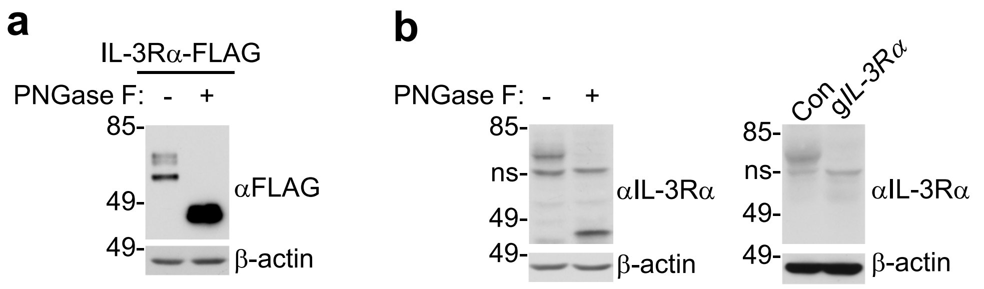

Supplementary Fig. S1. IL-3Rα is glycosylated.
a HEK 293 cells were transfected with C-terminal FLAG-tagged IL-3Rα plasmid for 20 hours. The cell lysates were treated with PNGase F or left untreated before immunoblotting analysis with anti-FLAG or anti-b-actin.
b Lysates of TF-1 cells were treated with PNGase F or left untreated before immunoblotting analysis with anti-IL-3Rα or anti-β-actin (left blots). Lysates of control or IL-3Rα knockout (g*IL-3Rα*) TF-1 cells were analyzed by immunoblots with anti-IL-3Rα or anti-β-actin, which served as a control for the specificity of the anti-IL-3Rα antibody. ns, non-specific band.

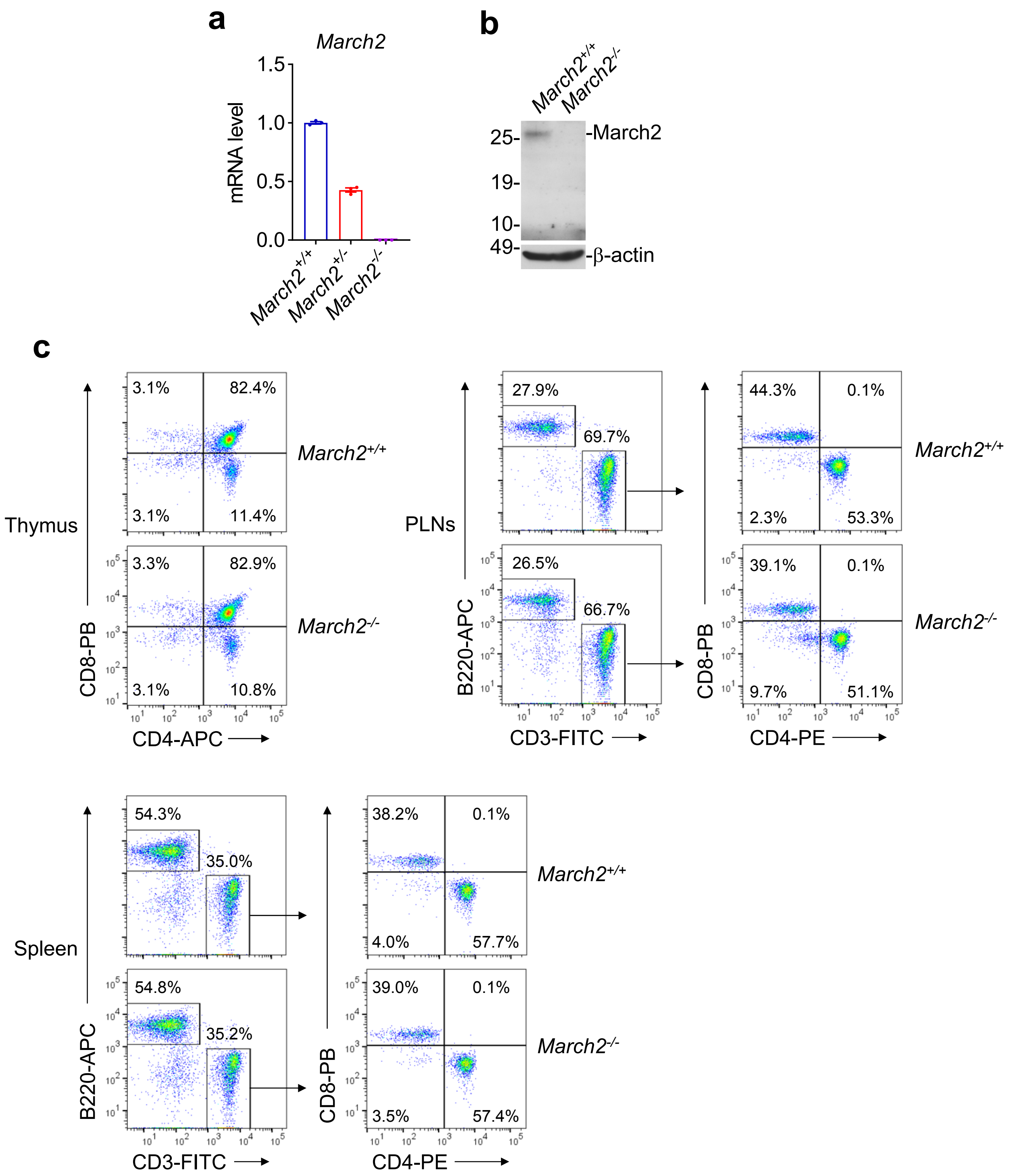

Supplementary Fig. S2. Analysis of March2-deficient mice.

a qPCR analysis of *March2* mRNA abundance in *March2*$^{+/+}$ and *March2*$^{-/-}$ BMDMs.

b Immunoblotting analysis of March2 level in *March2*$^{+/+}$ and *March2*$^{-/-}$ BMDMs.

c March2-deficiency has no marked effects on differentiation and proliferation of T and B cells. Cells from thymus, PLNs and spleen of sex- and age- matched *March2*$^{+/+}$ and *March2*$^{-/-}$ mice were analyzed by flow cytometry after staining with the indicated antibodies. Data shown are from one representative experiment, which was repeated for three times with similar results.

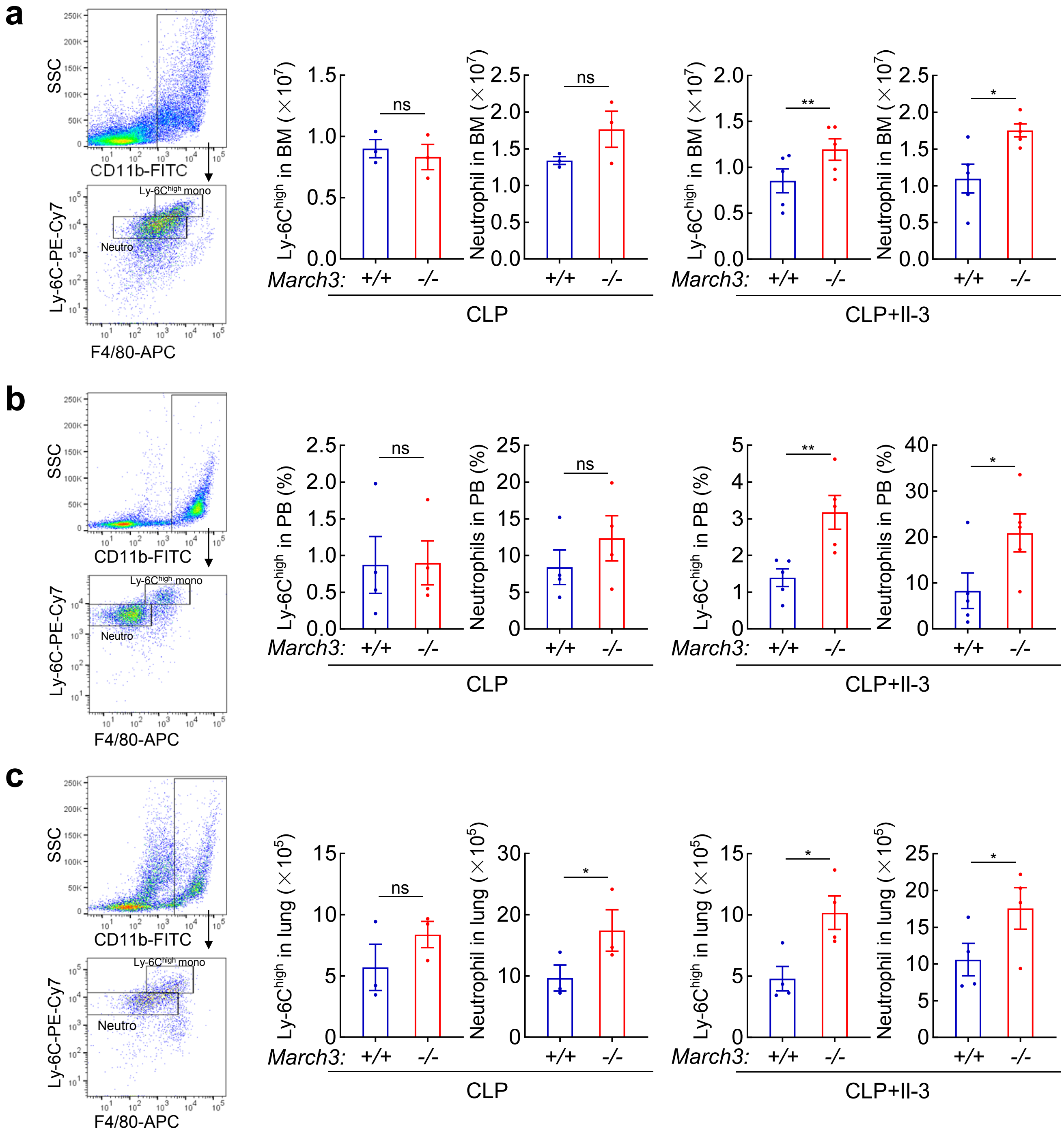

Supplementary Fig. S3. Ly-6C^{high} monocytes and neutrophils are increased in *March3$^{-/-}$* mice after CLP and IL-3 administration.

The mice were subjected to CLP and injected twice with Il-3 (3 mg each mouse) or PBS via tail vein at 30 min and 12 h after CLP and were sacrificed at 24 h before the following experiments.

a Numbers of Ly-6C^{high} monocytes and neutrophils in the bone marrow (BM). Gating strategy was shown at the left. Data shown are mean ± SEM, n=3 (CLP groups) or n=5 (CLP+Il-3 groups). * $P < 0.05$, ** $P < 0.01$; ns, not significant.

b Percentages of Ly-6C^{high} monocytes and neutrophils in the peripheral blood (PB). Gating strategy was shown at the left. Data shown are mean ± SEM, n=4 (CLP groups) or n=5 (CLP+Il-3 groups). * $P < 0.05$, ** $P < 0.01$; ns, not significant.

c Numbers of Ly-6C^{high} monocytes and neutrophils in the lung. Gating strategy was shown at the left. Data shown are mean ± SEM, n=3 (CLP groups) or n=4 (CLP+Il-3 groups). * $P < 0.05$; ns, not significant.
